# Digital Adoption in Paramedicine: A Scoping Review Protocol

**DOI:** 10.12688/hrbopenres.14229.1

**Published:** 2025-08-28

**Authors:** Brian McKeon, Dr. Eoin Coughlan, Dr. Siobhan Masterson, Dr. Karen Neville, Prof. Conor Deasy

**Affiliations:** 1 Department of Emergency Medicine and Pre-Hospital Care, University College Cork School of Medicine, Brookfield, College Road,, University College Cork, T12 AK54, Ireland; 2 National Ambulance Service of Ireland, Rivers Building, Tallaght, Dublin, D24 XNP2, Ireland; 3 School of Medicine, University of Galway, Clinical Science Institute, Discipline of General Practice, University of Galway, H91 TK33, Ireland; 4 Cork University Business School, Business Information Systems, University College Cork, O'Rahilly Building, College Road., Cork, Ireland; 5 Department of Emergency Medicine, Cork University Hospital, Wilton, Cork, T12 DFK4, Ireland

**Keywords:** Paramedicine, Paramedic, Emergency Medical Technician, EMT, Emergency Medical Services, Digital Acceptance, Digital Adoption, Technology

## Abstract

**Background:**

Since the late 1980s there’s been a growing body of knowledge that applies behavioural and cognitive theories to understanding how people accept new technologies. However, a paradox exists between studies demonstrating the many benefits of Digital Systems in Healthcare and the volume of literature detailing end-user clinician acceptance issues. The problems encountered when designing, developing, and operating complex technologies are not limited to technical or engineering issues. Technology implemented in organizations does not stand alone but is part of a broader socio-technical system, spanning divergent groups of people, each with their own cultural dynamics and distinct ways of working. Paramedics have personality traits that may be advantageous in their career, but research is needed to determine how these affect their ability to accept new technologies.

**Methods:**

This scoping review will be conducted according to Joanna Briggs Institute (JBI) guidelines has been registered with the Open Science Foundation (OSF). Electronic searches for relevant publications will be conducted in MEDLINE, PubMed, Scopus, CINHAL, IEEE, ACM, PsycINFO, Web of Science. Global English language literature related to digital acceptance in Emergency Medical Services after 2004 will be included.

**Results:**

The process of extraction, analysis, and presentation will be conducted using the Preferred Reporting Items for Systematic Reviews and Meta-Analyses extension for Scoping Reviews (PRISMA-ScR).

**Conclusions:**

This scoping review will collect meta-data on Behavioural, Cognitive, and Environmental factors common to the dominant prevailing digital acceptance theories to determine if any would be effective in explaining the experience of implementing technology with Paramedics and Emergency Medical Technicians (EMTs) working in Emergency Medical Services.

## Introduction

### Digital acceptance in paramedicine

Since the late 1980s there’s been a growing body of knowledge that applies behavioural and cognitive theories to understanding how people accept new technologies. Models were developed, and have been applied to healthcare in general, but an initial search would indicate that there is limited research into the socio-technical, cognitive, and behavioural factors that determine how Paramedics accept new technologies.

The goal of the proposed scoping review is to analyse the current literature on digital acceptance in Paramedicine to determine if any prevalent models are appropriate to form the basis for future research.

### Impact of new technologies in healthcare

There are many benefits to the implementation of technology in commercial organizations, including productivity growth, organizational expansion, efficiency, effectiveness, competitiveness etc. ^1^. In healthcare organizations, the implementation of technology leads to quantitative benefits through increased revenue or averted costs. It can also lead to many qualitative benefits including improved quality of care, patient safety, and improved coordination of care ^2^.

Despite an abundance of literature pertaining to the benefits of technology in healthcare, there are also many studies that describe difficult implementations. Topaz *et al*. (2017) surveyed 469 nurses from 45 countries and found that two thirds of participants reported their current recording systems are not suitable for nursing clinical practice ^3, 4^. Software systems have been linked to physician burnout ^5^ and Doctors describe how these technologies have created a ”...monster of incomprehensibility” ^6^. Design and features fail to cross cultural boundaries and there is a limited number of major international companies that dominate competitive processes ^7^. A paradox exists between studies demonstrating the many benefits of Digital Systems in Healthcare ^1, 2^ and the volume of literature detailing end-user clinician acceptance issues ^3– 6^.

### Socio-technical systems in healthcare

The problems encountered when designing, developing, and operating complex technologies are not limited to technical or engineering issues. Technology implemented in organizations does not stand alone but is part of a broader socio-technical system, spanning divergent groups of people, each with their own cultural dynamics and distinct ways of working ^8, 9^. For improved acceptance, systems engineering and technology implementation may benefit from an understanding of this broader socio-technical perspective.

Healthcare work is highly complex and takes place across primary, secondary and tertiary care sectors, in private or public settings. Users are from diverse professional groups and services, some in patient contact (including doctors, nurses, and allied health professionals) and others are indirectly involved (including health administrators, epidemiologists, health economists) ^10^. It is unsurprising that large-scale technology implementations (such as Electronic Health Records) which look to encompass broad aspects of healthcare encounter issues with end-user acceptance. To better understand individuals or groups behaviour, and the factors that impact successful technology implementations, researchers have created digital acceptance models.

***An Overview of Digital Acceptance Modelling***. Since the “Cognitive Revolution” ^11^ between the 1950s and 1970s, with the shift in focus to internal mental processes and developments in the understanding of information processing, cognitive theories have been the bedrock of the development of technology acceptance models. Fishbein & Ajzen’s Theory of Reasoned Action (1975) and Bandura’s Social Cognitive Theory (1977) became the foundation for the development of the first technology acceptance models ^12, 13^.

In the 1980s there was limited empirical insight into users' involvement in the design and implementation of information systems. In 1989 Davis embarked on the development of a Technology Acceptance Model (TAM) by framing the processes mediating the relationship between information system characteristics and actual system use ^14^. According to TAM, acceptance is a three-stage process, whereby external factors (system design features) trigger cognitive responses (”Perceived Ease of Use” and ”Perceived Usefulness”), which in turn form an affective response (attitude toward using technology/intention), influencing Use Behaviour ^14, 15^.

Further work by Venkatesh and Davis in 2000, determined that ”Perceived Usefulness” was the strongest predictor of intention to use. They investigated the key antecedents of ”Perceived Usefulness” and created TAM2 which incorporated new constructs and moderators: subjective norm, image, job relevance, output quality, result demonstrability, experience, and voluntariness ^16^.

Subsequent to this Venkatesh identified a number of limitations with TAM, TAM2 and its variants. He analysed all the dominant technology models and created the Unified Theory of Acceptance and Use of Technology (UTAUT). This unified model suggested that the actual use of technology is determined by behavioural intention. The perceived likelihood of adopting the technology is dependent on the direct effect of four key constructs: Performance Expectancy, Effort Expectancy, Social Influence, and Facilitating Conditions ^17^.

Venkatesh continued to refine and develop UTAUT in response to critiques or in the course of pursuing new research opportunities. The limited external validity of the model drove further research to add additional determinants of behaviour, such as trust, self-efficacy, computer self-efficacy, innovativeness, perceived threats, and perceived risk ^18, 19^. The model was also extended by introducing new moderating effects, such as income, location, culture, and technology readiness ^20, 21^.

TAM, UTAUT, and their variants are some of the most established models in literature ^22^. The models and variants evolved from theories with common antecedent cognitive, behavioral, and environmental factors. Therefore, to determine if one of these models significantly explains the experience implementing technologies for Paramedics in EMS organizations, it is proposed to collect meta-data on factors common to all models, including their positive or negative impact on digital acceptance.

### Understanding paramedic behavioural and cognitive traits

When researching how digital acceptance models apply to Paramedicine, it’s not only important to understand the constructs and moderating factors of the models, but also particular behavioural or cogitative traits specific to Paramedics. Globally, EMS have a broad range of clinicians who provide pre-hospital treatment to stabilize and transport patients to appropriate secondary or tertiary healthcare facilities. In Ireland, the vast majority of EMS services are Paramedic-provided, with a small proportion of EMTs also providing direct patient care.

Paramedics have been shown to “display the ability to problem solve, critically analyse, perform complex reasoning, and work closely with the patient as well as in a group. They are adept at rapidly forming clinical impressions in the critically ill with minimal information, and are able to modulate their interventions accordingly, while simultaneously continuing to gather data as they perform life-saving measures” ^23^. Using a modified version of the Hamburg Personality Inventory (HPI), Pajonk *et al*. (2010) found that Paramedics score high in Contentiousness, Sensation Seeking, Resiliency, and Empathy. They have also been shown to score low in Extroversion, and Neuroticism (important in mitigating burnout) ^24, 25^. These personality traits may be advantageous in their career, but further research is needed to examine how they affect the ability to accept new technologies, which may require different skills and expertise from their core profession.

## Aims and objectives

It is proposed to conduct a scoping review of literature related to the implementation of Technology in EMS. The primary goal of the review is:

*To determine if any of the current models of Digital Acceptance would be effective for Paramedics or EMTs working in Emergency Medical Services.*

### The rationale for conducting a scoping review

Scoping Reviews are conducted when it may be difficult to produce a critically appraised and synthesized answer to the question ^26^. Preliminary searches have found a paucity of literature related to Digital Acceptance in Paramedicine.

Scoping Reviews are also used to identify common characteristics or concepts within the literature ^27^. Analysing the literature using the key concepts underpinning the Behavioural, Cognitive, and Environmental roots of mainstream Digital Acceptance Models may elicit a general compatibility with existing models or alternatively allow us to understand where current models are deficient with the domain of paramedicine.

### Rationale for limiting population to paramedics and EMTs

In Europe, and globally, there is a wide variety in the clinical specialities in an Emergency Ambulance who respond to calls ^28^. The aim of the proposed scoping review is to determine if a digital acceptance model can be found that could be researched in the National Ambulance Service of Ireland. As Ireland is predominantly a Paramedic and EMT based service, we will limit the search to sources that describe technology implementation for those specialities. Pajonk *et al*. found that ”the personality types of emergency physicians and paramedics are not homogeneous” ^25^. A scoping review conducted by Mason *et al*., 2020, discovered conflicting results when attempting to ascertain if Nurses (including Emergency Nurses) and Paramedics had similar dominant personality types ^29^. Therefore, this scoping review will be limited to Paramedics and EMTs to remove any confounding data introduced by other professions.

## Methods

This scoping review will be conducted according to Joanna Briggs Institute (JBI) guidelines ^27^ has been registered with the Open Science Foundation (OSF).

### Eligibility criteria

The PCC Framework (population, concept, and context) is “recommended by JBI as a guide to construct clear and meaningful objectives and eligibility criteria” ^30^.

- Population:
  ○ Paramedics and EMTs
- Concept:
  ○ Studies that describe or evaluate Technology implementations (not proposed or theoretical) that affect clinical practice, experience, or perception of Technology that is used in relation to patient care.
- Context:
  ○ Paramedics and EMTs in Emergency, Community Paramedicine, or Pre-Hospital Care Services, in any country, run by an Emergency Medicine Service

### Search method

Electronic searches for relevant publications will be conducted in MEDLINE, PubMed, Scopus, CINHAL, IEEE, ACM, PsycINFO, Web of Science. The databases and search strings were designed with the assistance of an expert librarian (see Table 1).

**Table 1.** Search Strategy.

**Population**	(Paramedic or Paramedicine or Paramed* or EMT or “Emergency Medical Technician”)
**Concept**	(“Emergency Medical Service” or EMS or Ambulance or Helicopter or "Critical Care" or Retrieval or "Rapid Response" or "Alternative Pathway") AND (Digital or Adoption or Computer or Technology or Telemetry or Device or App* or Software or Hardware) AND (Adoption or Change or Transition or Upgrade or Implementation or Project)
**Context**	Year > 2004 English Language

### Screening

All references will be imported into reviewing software and duplicates removed. The primary reviewer will screen the title and abstract in accordance with the eligibility criteria. Independent screening will be carried out by the second reviewer, with any disagreements discussed and resolved by the third reviewer. 

Grey literature (conference abstracts, government reports, private company reports, web articles etc.) will not be included in the review. The aim of the scoping review is to determine common characteristics or concepts in the literature, so there is a significant risk that the effect of publication (and other) bias from grey literature would confound the data ^31^.

There is a large variance in the number and structure of EMS organizations in different countries. EMS may be public or private, national or regional, and have different protocols and standards ^28^. As each organization generally serves a large population, there are a limited number of EMS globally, therefore English language papers from any country will be included in the scoping review.

### Data extraction

The process of extraction, analysis, and presentation will be conducted using the Preferred Reporting Items for Systematic Reviews and Meta-Analyses extension for Scoping Reviews (PRISMA-ScR) ^30^. Data will be extracted from all sources by the primary reviewer. The second reviewer will perform an audit of 10% of the sources. The third reviewer will perform an audit of 10% of the sources (excluding the second reviewers’ sources).

### Data synthesis

Data will be populated using the following metadata fields:

- Year
- Country
- Population in Study
  ○ Staff Type
    ▪ Paramedic
    ▪ Advanced/Specialist Paramedic
    ▪ Emergency Medical Technician
    ▪ Combination of staff types
  ○ Number of staff studied

- Technology Type (Software, Device, Hardware, All)

The following headings will be analysed in terms of their positive (+) or negative (-) impact on Technology Acceptance.

- Constructs:
  ○ Performance Expectancy (+/-)
    ▪ *Including: Perceived Usefulness, Extrinsic Motivation, Job-Fit, Relative Advantage, Outcome Expectations*
  ○ Effort Expectancy (+/-)
    ▪ *Including: Perceived Ease of Use, Complexity, Ease of Use*
  ○ Social Influence (+/-)
    ▪ *Including: Subjective Norm, Social Factors, Image*
  ○ Facilitating Conditions (+/-)
    ▪ *Including: Perceived Behavioural Control, Facilitating Conditions, Compatibility*
  ○ Organisational Environment (+/-)
    ▪ *Including: Macro Organisational Change, National Policy, Industrial Relations Environment*
  ○ Paramedic Traits (+/-)
    ▪ *Including: High: Contentiousness, Sensation Seeking, Resiliency, Empathy. Low: Extroversion and Neuroticism*

- Moderators:
  ○ Gender
  ○ Age
  ○ Experience
  ○ Voluntariness of Use

### Protocol registration

This protocol has been registered with the Open Science Framework (OSF)

### Study status

The current status of the review is that formal screening of search results against the eligibility criteria is ongoing.

## Data Availability

No data is associated with this article. Not applicable
