# Supplementary material for: Digital Adoption in Paramedicine: A Scoping Review Protocol
Source: HRB Open Res. 2025 Aug 28;8:96. [Version 1] doi: 10.12688/hrbopenres.14229.1 (PMC12775654; doi:10.12688/hrbopenres.14229.1)
Supplement: Peer review history

# Reviewer report: Reviewer response for version 1

**DOI:** 10.21956/hrbopenres.15650.r52429

**Published:** 2026-01-16

**Authors:** Kimberley Davis

**Affiliations:** 1 NSW Ambulance, New South Wales, Australia

Given the rapidly-evolving digital health environment, this represents an important body of work which may inform jurisdictional ambulance service implementation of new technologies amongst their workforce.

 Introduction

- The technology being discussed is not really defined. What kinds of things are the authors talking about? The main one it seems is eMR.
- EMT is provided as an acronym by not defined in-text. Obvious to those in the field but not elsewhere. Same with EMS.
- Have the authors defined the environment? Prehospital, critical care, or other relevant retrieval terminology is not used. Please expand.

Methods

- Overall Methods are sound; limiting to paramedics/EMTs rather than other clinician types is appropriate as the authors outline.
- Why after 2004? Presumably not including 2004?
- Population – what about community or volunteer paramedics? Are road only paramedics/services being evaluated, what about aeromedical retrieval?

Is the study design appropriate for the research question?

Yes

Is the rationale for, and objectives of, the study clearly described?

Partly

Are sufficient details of the methods provided to allow replication by others?

Yes

Are the datasets clearly presented in a useable and accessible format?

Yes

Reviewer Expertise:

Prehospital care, Ambulance service, health service research

I confirm that I have read this submission and believe that I have an appropriate level of expertise to confirm that it is of an acceptable scientific standard, however I have significant reservations, as outlined above.

# Reviewer report: Reviewer response for version 1

**DOI:** 10.21956/hrbopenres.15650.r51780

**Published:** 2026-01-06

**Authors:** Nikiah Nudell

**Affiliations:** 1 The Paramedic Foundation, Duluth, USA

This is an interesting question that should yield results that are of interest. The authors should consider including an additional metadata field to capture the domain of practice (it does not appear that the organizational environment is intended for this purpose). Technology used in daily life (e.g., mobile phones) may have different implications than clinical equipment that is highly regulated (e.g., defibrillators), which may then differ from technology used for operations or decision-making.

Is the study design appropriate for the research question?

Partly

Is the rationale for, and objectives of, the study clearly described?

Yes

Are sufficient details of the methods provided to allow replication by others?

Yes

Are the datasets clearly presented in a useable and accessible format?

Yes

Reviewer Expertise:

paramedicine, machine learning, public policy

I confirm that I have read this submission and believe that I have an appropriate level of expertise to confirm that it is of an acceptable scientific standard, however I have significant reservations, as outlined above.

# Reviewer report: Reviewer response for version 1

**DOI:** 10.21956/hrbopenres.15650.r52430

**Published:** 2026-01-06

**Authors:** Dylan A Mordaunt

**Affiliations:** 1 Victoria University of Wellington, Wellington, New Zealand

This protocol outlines a scoping review of studies on technology implementation in EMS involving paramedics and EMTs, aiming to assess whether existing technology acceptance models adequately explain acceptance in paramedicine and to identify common constructs and moderators reported in the literature. 

 Strengths

 - Clear aim and justification, including rationale for focusing on paramedics/EMTs. 

 - Methods broadly align with JBI scoping guidance and include duplicate screening with arbitration. 

 - Intention to report using PRISMA-ScR is stated. 

 Major

 - The eligibility section says “Year > 2004” but doesn’t specify the earliest included year in plain terms.

 - Table 1 provides the concept structure, but replication requires full strings per database (fields, subject headings, limits).

 - Consider adding “prehospital/pre-hospital/out-of-hospital” terms** (or justify exclusion). This is a credible sensitivity issue for capture. It would be worth referencing any reported hedges.

 - Fix minor indexing/clarity issues (e.g., “CINHAL” should be “CINAHL”). 

 - It's worth considering PRESS for peer review of the search strategy.

 - Using PRISMA-P adds to quality and reproducibility.

Is the study design appropriate for the research question?

Yes

Is the rationale for, and objectives of, the study clearly described?

Yes

Are sufficient details of the methods provided to allow replication by others?

Partly

Are the datasets clearly presented in a useable and accessible format?

Not applicable

Reviewer Expertise:

Health economics, digital health, out-of-hospital, health services

I confirm that I have read this submission and believe that I have an appropriate level of expertise to confirm that it is of an acceptable scientific standard, however I have significant reservations, as outlined above.

# Reviewer report: Reviewer response for version 1

**DOI:** 10.21956/hrbopenres.15650.r50091

**Published:** 2025-11-25

**Authors:** Verity F Todd

**Affiliations:** 1 Auckland University of Technology, Auckland, Auckland, New Zealand

Title: Digital Adoption in Paramedicine: A Scoping Review Protocol

 General comments:

 This protocol is of a high standard, with comprehensive referencing and meticulous attention to detail. The protocol is appropriately supported by established frameworks (PRISMA) and the review has been registered with the Open Science Framework, demonstrating commitment to transparent and reproducible research practices.

 The aim of this study is to conduct a scoping review to determine whether current digital acceptance models are suitable for application to paramedics in emergency medical services.

 Is the rationale for, and objectives of, the study clearly described?

 Yes. The authors have clearly articulated gaps in the existing literature and provided strong justification for this review. The background section effectively addresses two key aspects: current and emerging technologies in healthcare delivery, and the specific characteristics of paramedic practice that may influence technology adoption. This dual focus appropriately contextualises the research question.

 Is the study design appropriate for the research question?

 The scoping review methodology is well-suited to mapping the breadth of literature on digital acceptance models in paramedicine. The PCC (Population, Concept, Context) framework is appropriately applied to this review question.

 The search strategy appears comprehensive; however, I note that the search term "prehospital" (or "pre-hospital") does not appear to be included. Was this intentional? Given that much paramedicine literature uses prehospital care terminology, this may be worth considering to ensure comprehensive capture of relevant studies.

 I have verified that the OSF registration matches the details included in this protocol.

 Are sufficient details of the methods provided to allow replication by others?

 Yes. The methods are well-presented and contain sufficient detail to enable future replication. However, one detail requires clarification: What is the earliest year that will be included in the search? Specifying the temporal scope of the review would strengthen the reproducibility of the search strategy.

 Are the datasets clearly presented in a useable and accessible format?

 The data synthesis section is clearly presented, with comprehensive descriptions of the technology acceptance themes that will be reported, including specific terms of interest. The planned approach to data extraction and charting is appropriate for a scoping review of this nature.

 Recommendation:

 This is a well-designed protocol that requires only minor clarification regarding:

 1.    The temporal scope of the search (earliest year to be included)

 2.    Consideration of whether "prehospital"/"pre-hospital" terminology should be included in the search strategy

Is the study design appropriate for the research question?

Yes

Is the rationale for, and objectives of, the study clearly described?

Yes

Are sufficient details of the methods provided to allow replication by others?

Yes

Are the datasets clearly presented in a useable and accessible format?

Yes

Reviewer Expertise:

prehospital

I confirm that I have read this submission and believe that I have an appropriate level of expertise to confirm that it is of an acceptable scientific standard.
